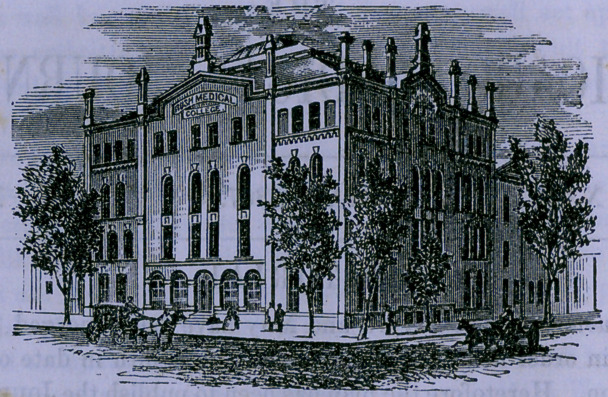# Rush Med. Col.—New Building

**Published:** 1867

**Authors:** 


RUSH MEDICAL COLLEGE—NEW BUILDING.

The new edifice for the College occupies nearly the whole previously vacant space between the old building and Indiana street, covering 4,200 square feet of surface, and fronting on Indiana street. It thus presents a frontage of seventy feet on that street, extending back to, and connecting with, the old College, at a depth of sixty feet. The latter structure covers about 3,000 square feet of surface, so that the whole area covered will be something over 7,000 square feet.

The old building, preserved on account of its massive and excellent foundation and walls, is being entirely reconstructed to conform to the general plan adopted.

The new edifice will contain two lecture rooms, with six hundred and twenty-three numbered seats in each. The lower lecture room is arranged as a semi-amphitheatre, with the seats rising in an easy grade in the usual manner. By removing a portion of the north wall of the old building, and the introduction of large sliding doors, this room opens into the laboratory, 31 by 32 feet. Through this latter opening a large table is readily moved upon a track into the area, at the pleasure of the lecturer. The laboratory will be supplied with all the modern appliances, and its ventilation is thoroughly provided for.

East of the laboratory there will be a small private lecture, demonstration, or "green room," with seats for about fifty students.

In front and under the grade of the lower lecture room, are several rooms adapted for the use of the Dispensary department, Professors' private apartments, closets, &c., &c.

The upper or anatomical lecture room affords a nearly complete amphitheatre, varying only in that, instead of completing the circle, the curved lines of the seats, at a little past the centre, are extended on each side to the rear wall, forming the letter U. This particular arrangement affords the Lecturer a large arena for his demonstrations, and from its rear there is direct communication with the Museum, so that the means of illustration can be always at command.

This lecture room will be very effective in its appearance, as it will be finished, open to the roof, showing the timbering and iron construction, which is entirely new in style and design. In the centre of the roof there will be a large sky-light, tastefully designed and finished.

There are regular windows on three sides of each of the large lecture rooms, affording ample light and ventilation.

The intermediate space between the upper and lower lecture rooms will be finished off with suits of Professors' rooms, with all the modern appliances to facilitate demonstration.

The Museum, occupying the front of the old building with the exception of the upper story, will be beautifully arranged with galleries, surrounded by glass cases, and connected by stairways, the upper gallery communicating, as before noted, with the upper lecture room. The main entrance to the Museum will be from Dearborn street, and from any point in the interior all parts can be taken at a *coup d'œil.*

The general dissecting room extends over the entire upper portion of the old College building, having thus an area of about 3000 square feet. It is magnificently lighted by two large skylights and side windows upon the south and west. It is provided with numerous closets, water, gas, &c., &c., is easy of access, and thoroughly ventilated. The Demonstrator's room adjoins it upon the south-east corner of the new building, so that every part of it may be under his constant and ready supervision.

The main entrance to the College will be on Indiana street. Besides this there will be two private entrances, one from Dearborn street for the Faculty, and which also communicates with that to the Museum; the other on Indiana street for the Janitor's rooms, as he is to reside in the building.

The College Free Dispensary will be reached by the main entrance.

Four distinct sets of stairways rise to every part of the entire structure, affording ample means of ingress and egress.

The fronts on Dearborn and Indiana streets will be finished with red pressed brick with stone trimmings, including cornices and chimney tops.

The front represents four stories, but the lecture rooms occupy the entire height of the building, the Professors' rooms, &c., finishing so as only to use the intervening space afforded by the grade of the seats.

There will be a cellar for fuel and for the heating apparatus. The system of heating will be by low pressure steam, by indirect radiation and ventilating air ducts all around the main lecture rooms.

It is scarcely necessary to remark, that the Faculty have sought, in planning the building, to take advantage of every improvement suggested by careful observation of the best constructed edifices of the kind, and have spared neither pains nor expense to make it a model structure. Their instructions to the architect, W. W. Boyington, Esq., so well known for the excellence of his architectural works throughout this city and the North-west, were fully carried out, and improved, under the guidance of his cultivated taste and great experience. The completed building will be unsurpassed for its purposes by anything of the kind in the country, and while supplying the wants of the College, will be seen to be an additional ornament to Chicago, which is fast becoming a city of palaces. The cut given at the head of this article very fairly represents the completed structure.

By the strict provisions of the contracts entered into with the builders, it is to be entirely finished on the fifteenth day of

September ensuing, and from the known and established reputation of the parties engaged, there is not the slightest apprehension of a day's delay beyond that period.

It is due to the Faculty to state, that the entire amount requisite for the construction of the College is provided from their own means, no contributions or assistance having been received or asked from any other quarter.

Total estimated cost, $75,000.

CONTRACTORS.

| | |
|---|---|
| *Masonry* | C. DAEGLING. |
| *Cut Stone* | MILLER & NEWCOMB. |
| *Carpentry* | T. C. BOYINGTON. |
| *Iron Work* | N. S. BOUTON & Co. |
| *Plumbing* | POWELL & PATTISON. |
| *Painting and Glazing* | J. W. WILSON. |
| *Sewerage* | C. GLADDING. |

---

*Practical Anatomy.*—Our readers have probably noticed in the secular press a strongly sensational account of the arrest of a couple of American citizens of African descent *in flagrante delictu*, having in possession several *cadavera* evidently designed for some anatomical theatre. It was supposed at first, from their proximity to the institution, that they were designed for the Rush College, but subsequent investigation showed, as was in fact the case, that in that iniquity, (if it be an iniquity,) Rush College, or any of its Faculty, had no part or lot. The morning dailies touched upon the affair merely as a matter of news, without any effort at a sensation, or to inflame the ignorant prejudices of the populace. But the evening sheets ascended to the highest peak of vituperative invective, and descended to the lowest depths of hoarse scurrility in their comments upon the matter. Everything was said that could be said to excite a mob spirit, but, fortunately, the Chicago public are too thoroughly accustomed to such diatribes to rise under the plentiful ferment, and in their great wrath smite things incontinently. As imaginative exercises are necessary to secure the sale of the evening prints, this course might be passed over without

## Figures and Tables

**Figure f1:**